# Polymeric Nanoparticles: Production, Characterization, Toxicology and Ecotoxicology

**DOI:** 10.3390/molecules25163731

**Published:** 2020-08-15

**Authors:** Aleksandra Zielińska, Filipa Carreiró, Ana M. Oliveira, Andreia Neves, Bárbara Pires, D. Nagasamy Venkatesh, Alessandra Durazzo, Massimo Lucarini, Piotr Eder, Amélia M. Silva, Antonello Santini, Eliana B. Souto

**Affiliations:** 1 Department of Pharmaceutical Technology, Faculty of Pharmacy, University of Coimbra, Pólo das Ciências da Saúde, Azinhaga de Santa Comba, 3000-548 Coimbra, Portugal; zielinska-aleksandra@wp.pl (A.Z.); filipacarreiro.fc@gmail.com (F.C.); anamargaridaoliveira.99@gmail.com (A.M.O.); andreianeve@hotmail.com (A.N.); bapires2000@hotmail.com (B.P.); 2 Institute of Human Genetics, Polish Academy of Sciences, Strzeszyńska 32, 60-479 Poznań, Poland; 3 JSS College of Pharmacy, JSS Academy of Higher Education & Research, Ooty 643 001, Tamil Nadu, India; nagasamyvenkatesh@rediffmail.com; 4 CREA-Research Centre for Food and Nutrition, Via Ardeatina 546, 00178 Rome, Italy; alessandra.durazzo@crea.gov.it (A.D.); massimo.lucarini@crea.gov.it (M.L.); 5 Department of Gastroenterology, Dietetics and Internal Diseases, Poznan University of Medical Sciences, Przybyszewskiego 49, 60–355 Poznań, Poland; piotr.eder@op.pl; 6 Department of Biology and Environment, University of Tras-os-Montes e Alto Douro, UTAD, Quinta de Prados, 5001-801 Vila Real, Portugal; amsilva@utad.pt; 7 Centre for Research and Technology of Agro-Environmental and Biological Sciences (CITAB-UTAD), Quinta de Prados, 5001-801 Vila Real, Portugal; 8 Department of Pharmacy, University of Napoli Federico II, Via D. Montesano 49, 80131 Napoli, Italy; 9 CEB—Centre of Biological Engineering, University of Minho, Campus de Gualtar, 4710-057 Braga, Portugal

**Keywords:** polymeric nanoparticles, nanocapsules, nanospheres, therapeutic potential, targeted drug delivery, toxicology, ecotoxicology.

## Abstract

Polymeric nanoparticles (NPs) are particles within the size range from 1 to 1000 nm and can be loaded with active compounds entrapped within or surface-adsorbed onto the polymeric core. The term “nanoparticle” stands for both nanocapsules and nanospheres, which are distinguished by the morphological structure. Polymeric NPs have shown great potential for targeted delivery of drugs for the treatment of several diseases. In this review, we discuss the most commonly used methods for the production and characterization of polymeric NPs, the association efficiency of the active compound to the polymeric core, and the in vitro release mechanisms. As the safety of nanoparticles is a high priority, we also discuss the toxicology and ecotoxicology of nanoparticles to humans and to the environment.

## 1. Introduction

**Figure 1:**
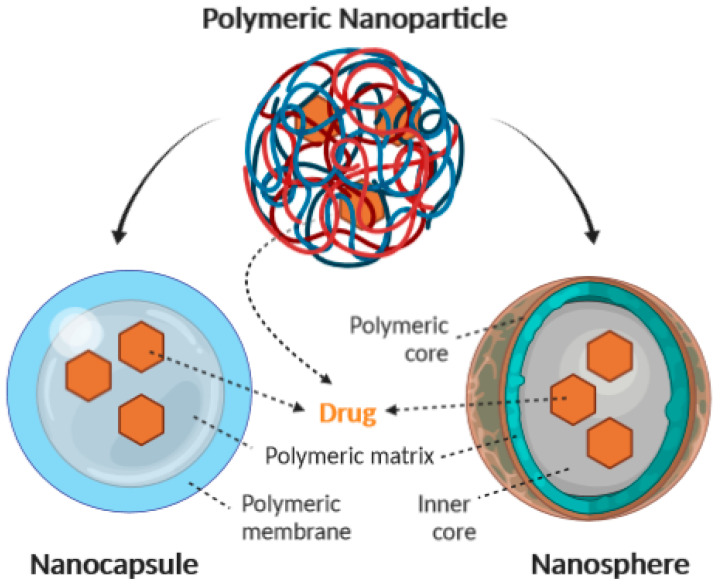
Schematic representation of the structure of nanocapsules and nanospheres (arrow stands for the presence of drug/bioactive within the nanoparticles).

Polymeric nanoparticles (NPs) have attracted considerable interest over recent years due to their properties resulting from their small size [1,2,3]. Advantages of polymeric NPs as drug carriers include their potential use for controlled release, the ability to protect drug and other molecules with biological activity against the environment, improve their bioavailability and therapeutic index [1,4]. The term “nanoparticle” comprises both nanocapsules and nanospheres, which differ with respect to their morphology [5]. Nanocapsules are composed of an oily core in which the drug is usually dissolved, surrounded by a polymeric shell which controls the release profile of the drug from the core. Nanospheres are based on a continuous polymeric network in which the drug can be retained inside or adsorbed onto their surface [5,6,7]. These two types of polymeric NPs recognized as a reservoir system (nanocapsule), and matrix system (nanosphere) [8] are shown in Figure 1. Examples of drugs/bioactive ingredients loaded in polymeric nanoparticles are depicted in Table 1.

## 2. Methods for Production of Polymeric Nanoparticles

Depending on the type of drug to be loaded in the polymeric NPs and their requirements for a particular administration route, different methods can be used for the production of the particles [26]. In general, two main strategies are employed, namely, the dispersion of preformed polymers or the polymerization of monomers [27,28]. Table 2 lists the most commonly used techniques [29,30].

In most of the techniques requiring the use of preformed polymers, organic solvents are commonly used in the first step to dissolve the polymer [28]. These solvents can generate problems related to toxicity and environmental risk. In addition, solvent residues must be removed from the final product. In order to load compounds in polymeric NPs, techniques based on the polymerization of monomers allow insertion with greater efficiency and in a single reaction step [31]. Regardless of the method of preparation employed, the products are usually obtained as aqueous colloidal suspensions [26].

### 2.1. Solvent Evaporation

**Figure 2:**
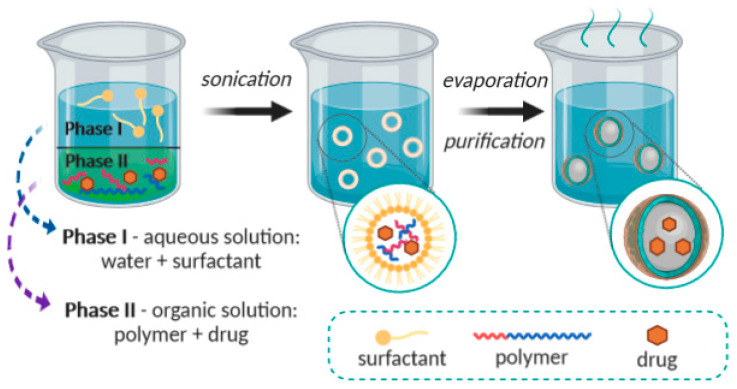
Schematic representation of the solvent evaporation method.

Solvent evaporation was the first method developed to prepare polymeric NPs from a preformed polymer. In this method, the preparation of an oil-in-water (o/w) emulsion is initially required [32], leading to nanospheres production [33,34]. The whole process is shown in Figure 2. Firstly, an organic phase is prepared, consisting of a polar organic solvent in which the polymer is dissolved, and the active ingredient (e.g., drug) is included by dissolution or dispersion. Dichloromethane and chloroform have been widely used, although more often in the past [35]. Due to their toxicity, they have been replaced by ethyl acetate [36], which displays a better toxicological profile, and therefore, it is more suitable for biomedical applications [37]. An aqueous phase, which contains a surfactant (e.g., polyvinyl acetate; PVA), has also been prepared frequently [36]. The organic solution is emulsified in the aqueous phase with a surfactant, and then it is typically processed by using high-speed homogenization or ultrasonication, yielding a dispersion of nanodroplets [38]. A suspension of NPs is formed by evaporation of the polymer solvent, which is allowed to diffuse through the continuous phase of the emulsion. The solvent is evaporated either by continuous magnetic stirring at room temperature (in case of more polar solvents) or in a slow process of reduced pressure (as happens when using e.g., dichloromethane and chloroform). After the solvent has evaporated, the solidified nanoparticles can be washed and collected by centrifugation, followed by freeze-drying for long-term storage. This method allows the creation of nanospheres [9].

### 2.2. Emulsification/Solvent Diffusion

**Figure 3:**
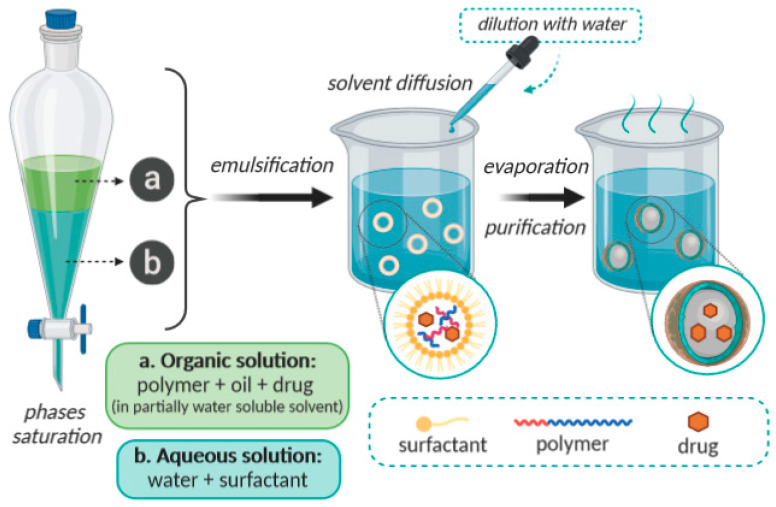
Schematic representation of the emulsification/solvent diffusion method.

This method consists on the formation of an o/w emulsion between a partially water-miscible solvent containing polymer and drug, and an aqueous solution with a surfactant [39,40]. The internal phase of this emulsion consists of a partially hydro-miscible organic solvent, such as benzyl alcohol or ethyl acetate, which is previously saturated with water in order to ensure an initial thermodynamic balance of both phases at room temperature [41]. The subsequent dilution with a large amount of water induces solvent diffusion from the dispersed droplets into the external phase, resulting in the formation of colloidal particles. Generally, this is the method used to produce nanospheres, but nanocapsules can also be obtained if a small amount of oil (such as triglycerides: C6, C8, C10, C12) is added to the organic phase. Finally, depending on the boiling point of the organic solvent, this latter stage can be eliminated by evaporation or by filtration [7]. This method is schematically shown in Figure 3. At the end, it possible to obtain NPs with dimentions ranging from 80 to 900 nm. This method is frequently applied for polymeric NPs production, despite the requirement of a high volume of the aqueous phase, which must be eliminated from the colloidal dispersion, and despite the risk of diffusion of the hydrophilic drug into the aqueous phase [42,43].

### 2.3. Emulsification/Reverse Salting-Out

**Figure 4:**
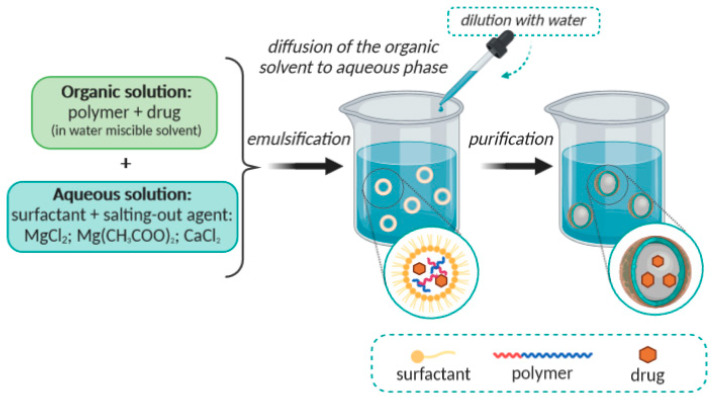
Schematic representation of the emulsification/reverse salting-out method.

The above described emulsification/solvent diffusion method can be considered as a modification of the emulsification/reverse salting-out method. The salting-out method is based on the separation of a hydro-miscible solvent from an aqueous solution, through a salting-out effect that may result in the formations of nanospheres [44]. The main difference is the composition of the o/w emulsion, which is formulated from a water-miscible polymer solvent, such as acetone or ethanol, and the aqueous phase contains a gel, the salting-out agent and a colloidal stabilizer [45]. Examples of suitable salting-out agents include electrolytes, such as magnesium chloride (MgCl_2_), calcium chloride (CaCl_2_) or magnesium acetate [Mg(CH_3_COO)_2_], as well as non-electrolytes e.g., sucrose [27,46]. The miscibility of acetone and water is reduced by saturating the aqueous phase, which allows the formation of an o/w emulsion from the other miscible phases [47,48]. The o/w emulsion is prepared, under intense stirring, at room temperature. Then, the emulsion is diluted using an appropriate volume of deionized water or of an aqueous solution in order to allow the diffusion of the organic solvent to the external phase, the precipitation of the polymer, and consequently, the formation of nanospheres. The remaining solvent and salting-out agent are eliminated by cross-flow filtration. The condition of complete miscibility between the organic solvent and water is not essential but it simplifies the execution process [44,49]. This method is schematically presented in Figure 4. The dimensions of the nanospheres obtained by this method vary between 170 and 900 nm. The average size can be adjusted to values between 200 and 500 nm, by varying polymer concentration of the internal phase/volume of the external phase [6].

### 2.4. Nanoprecipitation

**Figure 5:**
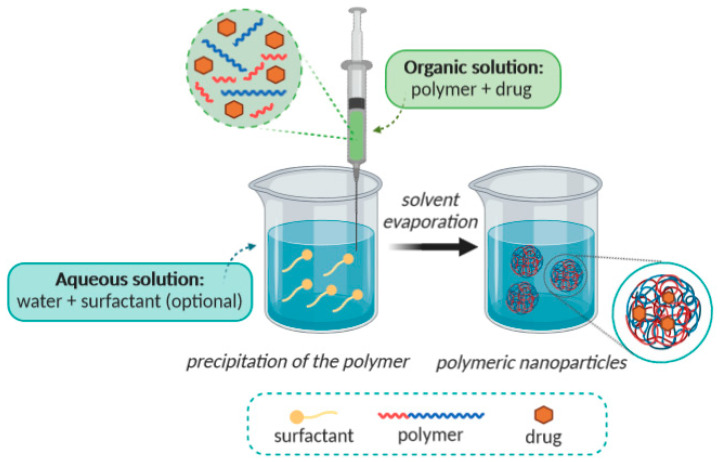
Schematic illustration of the nanoprecipitation method.

This method, also designated as solvent displacement method, requires two miscible solvents (Figure 5). The internal phase consists of a polymer dissolved in a miscible organic solvent, such as acetone or acetonitrile [50,51,52,53,54]. Because of immiscibility in water, they can be easily removed by evaporation. The principle of this technique relies on the interfacial deposition of a polymer after the displacement of the organic solvent from a lipophilic solution to the aqueous phase [55]. The polymer is dissolved in a water-miscible solvent of intermediate polarity, and this solution is added stepwise into an aqueous solution under stirring (in a dropwise way), or by controlled addition rate. Due to the fast spontaneous diffusion of the polymer solution into the aqueous phase, the nanoparticles form instantaneously in an attempt to avoid the water molecules [55]. As the solvent diffuses out from the nanodroplets, the polymer precipitates in the form of nanocapsules or nanospheres. In general, the organic phase is added to the aqueous phase, but the protocol can also be reversed without compromising the nanoparticle formation [56]. Usually, surfactants can be included in the process to guarantee the stability of the colloidal suspension, although their presence is not required to ensure the formation of nanoparticles. The obtained nanoparticles are typically characterized by a well-defined size and a narrow size distribution, which are better than those produced by the emulsification solvent evaporation procedure [57]. Nanoprecipitation is a method frequently used for the preparation of polymeric NPs with around 170 nm dimensions [58], but it also allows the acquisition of nanospheres or nanocapsules [7]. Nanospheres are obtained when the active principle is dissolved or dispersed in the polymeric solution. Nanocapsules are obtained when the drug is previously dissolved in an oil, which is then emulsified in the organic polymeric solution before the internal phase is dispersed in the external phase of the emulsion [55,56].

## 3. Characterization of Polymeric Nanoparticles

Polymeric NPs may differ in physical properties, such as composition and concentration, as well as in size, shape, surface properties, crystallinity, or in dispersion state. These properties are usually assessed by several methods, aiming for the full characterization of the NPs. Electron microscopy, dynamic light scattering (DLS) or photon correlation spectroscopy (PCS), Near-infrared spectroscopy, electrophoresis, and chromatography are a few of the most commonly used [59,60]. Polymeric NPs characterization is very important, in terms of its applicability, but also to ascertain issues concerning nanotoxicology and exposure assessment in workplaces, which are important to assess their health and safety hazards, as well as to control manufacturing processes [61].

### 3.1. Morphology

Scanning and transmission electron microscopy (SEM and TEM) have been widely used to obtain information regarding the shape and size of polymeric NPs. These are usually combined with cryofracture techniques to perform the NPs morphology analysis. TEM is widely used and is capable of distinguishing between nanocapsules and nanospheres, in addition to being able to determine the thickness of the nanocapsule wall [36]. Nanospheres have a spherical shape, with a solid polymeric structure, whereas nanocapsules are formed by a thin (about 5 nm) polymeric envelope around the oily core. Another technique that has been used to characterize the surface morphology of polymeric NPs is atomic force microscopy (AFM) [62]. It provides information with high resolution in three dimensions, and in a nanometric scale, while it is also able to resolve surface details at an atomic level [63]. By applying this technique a complex topography on the surface of the nanoparticles has been observed, while by analyzing sections of samples, the presence of small cavities and pores has also been revealed [6].

### 3.2. Particle Size Distribution

In general, polymeric NPs obtained from different methods may have mean diameters between 100 and 300 nm. The polydispersity should be as low as possible (ideally, nearly zero), and the size distribution unimodal. Particles with diameters around 60 to 70 nm or even less than 50 nm can also be obtained [64]. The nanoparticle size can be measured by using different techniques, the most commonly used being the dynamic (DLS) and static (SLS) light scattering, but TEM, SEM and AFM are also oftenly used [65]. Size measurements may vary depending on the method used, for example, electron microscopy provides an image of the particle isolated from the surroundings, while DLS allows the determination of the hydrodynamic radius of suspended particles. Moreover, DLS is an important complement to TEM, because it can measure larger sizes, providing information on the aggregation state of a nanoparticle in solution by determining changes in particle size distribution [65,66]. Many factors can influence the size of polymeric NPs, such as the quali-quantitative composition, a example is the case of nanocapsules, in which during their production a factor that influences the particle diameter is the nature of the oil used as the core, due to differences in viscosity, hydrophobicity or interfacial tension between the different liquid phases. Another factor that can influence the average diameter of the nanoparticles is the amount of drug that may lead to larger particles with wider size distribution [6,7].

### 3.3. Chemical Composition and Crystal Structure

Chemical composition refers to the atomic elements of which a nanoparticle is composed, as well as compounds native or formed functional groups, and it can be measured in an ensemble or single-particle elemental analysis method. One of the most common ensemble techniques used is atomic absorption spectroscopy which is based on the principle of atomic absorption, where ground state electrons of the atoms jump to an excited state by absorbing a certain quantity of energy from light at a specific wavelength [67]. Because the amount of energy absorbed is related to the type and the number of atoms in the light path, the sample mass concentration can be quantified by comparing the signal with calibration standards at known concentrations. One of the techniques used to determine the chemical composition of a single particle is time-of-flight mass spectrometry (TOFMS) that consists in ionizing small to large organic analytes into the gas phase with minimal fragmentation and their subsequent separation/detection using a time-of-flight mass analyzer [68]. The arrangement of elemental atoms in a nanoparticle may be organized into a crystal structure or it may be amorphous. Generally, crystal structure is determined using powder X-ray diffraction, or selected area electron diffraction using a transmission electron microscope. X-ray diffraction requires that about a gram of material is available for analysis, whereas electron diffraction can be done on single particles [69,70,71].

### 3.4. Molar Mass Distribution of the Polymer

The determination of the polymer molar mass distribution, after preparation, can provide information regarding the influence of formulation components on the polymerization process, the occurrence of chemical reactions between the drug and the polymer, and also regarding the degradation of the polymer [72]. The most commonly used technique for determining the polymer molar mass distribution is size-exclusion chromatography (SEC) [73,74]. Furthermore, static light scattering (SLS), has also been used to analyze the intensity of light spread by the polymeric NPs [66].

### 3.5. Surface Area and Chemistry

The NPs’s surface area is relevant due to its influence on reactivity and surface interactions with ligands. Different methods measure different aspects of surface area. The direct measurement of the nanoparticle’s surface area uses adsorption of an inert gas (such as N_2_) under varying conditions of pressure to form a monolayer of gas coverage [75]. The number of gas molecules that is necessary to form a monolayer and the cross-sectional area of the adsorbate gas molecule is related to the “total surface area”. This method is also used to evaluate morphology of porous materials, as the gas also binds to internal pores and crevices [75,76]. Surface chemistry refers to the elemental or molecular chemistry of a particle surface. For nanoparticles, a higher proportion of atoms are on their surfaces, (due to higher area/volume ratio) and these atoms are in direct contact with solvents and influence their interactions with other molecules [77]. Some nanoparticles, such as nanocapsules have a core-shell structure, in which the outer surface atoms are different from those of the interior core. Multiple techniques are available to characterize nanoparticle surface chemistry, for example X-ray photoelectron spectroscopy and secondary ion mass spectroscopy [78].

### 3.6. Zeta Potential

The zeta potential (ζ) reflects the surface charge of the particles, which is influenced by changes in the interface with the dispersing medium, due to the dissociation of functional groups on the particle’s surface or due to the adsorption of ionic species present in the aqueous dispersion medium as well as the solvation effect [79]. This parameter is determined using Doppler techniques to measure the particle velocity as a function of voltage, thus the zeta potential is calculated from the electrophoretic mobility of particles in a respective solvent [71,80]. Phospholipids, poloxamers, and polymers are the main components of polymeric NPs and, once present in formulations, are capable of influencing the zeta potential. A relatively high zeta potential value, considered as |± 30 mV|, is important for good physicochemical stability of the colloidal suspension, as large repulsive forces tend to prevent aggregation due to occasional collisions with adjacent nanoparticles [71]. The zeta potential determination is useful in elucidating the mechanism of association of drugs with nanoparticles [79,81]. Calvao et al. [82] reported that the zeta potential was instrumental to understand the loading of albumin into nanospheres produced from chitosan and a diblock complymer of ethlene oxide and propylene oxide (PEO-PPO). Calvo et al. have observed the effects of the composition of the different formulations on the values of zeta potential [82]. The zeta potential of NPs can thus be tailored for a specific application, by introducing surfactants or other coatings onto the NPs surface, such as poly-ethylene-glicol (PEG) of varying molecular weights [61,83].

### 3.7. pH of Suspensions

Relevant information on the stability of nanoparticulate suspensions can be obtained by monitoring pH as a function of time. For example, the changing of pH may indicate polymer degradation, as it implies changes in protonation at particles surfaces. In a work carried out by Calvo et al., a decrease in molar mass was verified in suspensions of nanocapsules and nanospheres, after 6 months of storage, with a consequent decrease in the pH of these formulations [7,82]. However, the decrease in the pH values of suspensions, in a short period of time, can be attributed both to the ionization of carboxylic groups, present in the polymer, releasing protons to the surrounding medium, depending on the hydrophobicity of the polymer. Additionally, the pH of the medium may influence the zeta potential and the electrostatic stability of formulation, thus its monitorization is of great relevance.

### 3.8. Stability of Polymeric NPs Suspensions

Colloidal suspensions usually do not tend to phase separation until a few months after preparation, because the sedimentation process is slow for submicrometric particles and even more minimized by the Brownian movement. However, particle agglomeration and sedimentation processes can occur over time [84]. Several factors can influence the stability of colloidal suspensions, such as the adsorption of active molecules on the surface of the nanoparticles and the presence of adsorbed surfactants. Some physicochemical parameters that can be used to monitor the stability of polymeric colloidal suspensions are particle size, zeta potential, polymer molar mass distribution, drug content, and pH [85]. However, industrial application of polymeric NPs dispersed in aqueous media can be limited due to problems of low physicochemical stability, in prolonged storage periods [86]. The main limitations are the particle aggregation, the polymer chemical stability, the drug, or other raw materials used during NPs production and also the premature release of the active substance. In addition, it is important to emphasize that liquid dosage forms are prone to microbial proliferation with the need to add preservatives [87]. In order to delay or avoid these physicochemical and microbiological problems, drying, such as lyophilization (freeze-drying) or spray drying is usually recommended. Lyophilization consists of removing water through sublimation and has been widely used for drying nanosphere suspensions [88]. On the other hand, spray drying as an alternative to lyophilization, with the objective of increasing the stability of nanoparticles formed by solid lipids, consists of passing the solution through an atomizing orifice, into the drying chamber in the form of droplets, in co-current, counter-current or mixed flow of hot air, which promotes the rapid drying of the droplets. The dry solid particles are then separated and collected and can be presented in the form of fine powders, granules, or agglomerates [89,90].

### 3.9. Determination of the Drug Association

**Figure 6:**
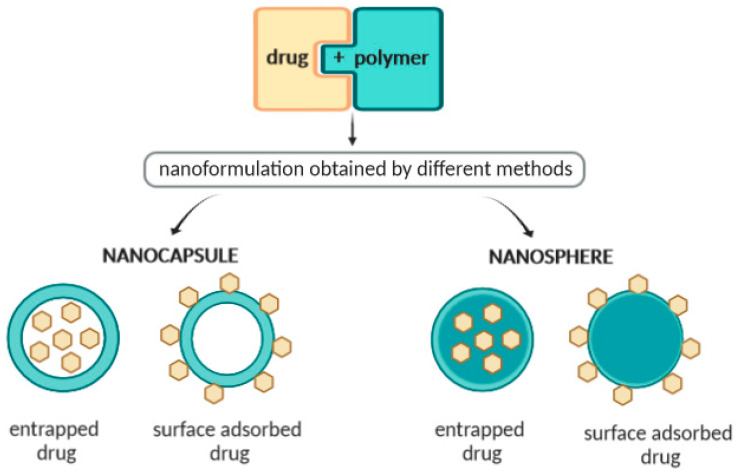
Different possibilities of the drug association with nanospheres and nanocapsules.

Determination of the amount of drug associated with nanoparticles is especially complex due to their small size, which makes it difficult to separate the free fraction of the drug from the associated fraction [91]. A widely used separation technique is ultracentrifugation, in which the free drug, present in the suspension, is determined in the supernatant after centrifugation. The total drug concentration is usually determined by the complete dissolution of a fraction of the nanoparticles in a suitable solvent. Therefore, the concentration of drug associated with the nanosparticles is calculated by the difference between the total and the free drug concentrations [92,93]. Another method that has also been used is the ultrafiltration-centrifugation, in which a membrane is used to separate part of the dispersing aqueous phase from the colloidal suspension. The free drug concentration is determined in the ultrafiltrate, and the drug fraction associated with the nanostructures is also calculated by subtracting the total and free concentrations [92]. According to published studies, several factors may influence the amount of drug associated with nanostructured systems, such as: physicochemical characteristics of the drug, pH of the medium, NPs surface characteristics or nature of the polymer, the amount of drug added to the formulation, the order of addition of drug to the formulation (before or after the formation of nanostructures), nature of the oil used (in the case of nanocapsules), as well as the type of surfactant adsorbed to the polymeric surface [94,95,96]. By modifying the particles’ surface characteristics, it is possible to obtain different rates of drug association by adsorption, for the same initial drug concentration. This parameter is very important to determine the ability to prolong the drug’s action time. Therefore, it is relevant to determine the drug adsorption isotherm on the surface of the nanoparticles, since it provides information on how the drug is distributed on the particle surface and its association capacity [95]. Regarding the nanospheres, different forms of drug association are described; the drug may be dissolved or dispersed within the polymeric matrix, or may be adsorbed to the polymer. Nanocapsules are, on the other hand, produced to increase the loading of lipophilic drugs, which should be entrapped by the polymeric membrane dissolved in the oily core (Figure 6) [7].

Determination of drug association mode is also a complex procedure since the available methods only determine the concentration of the drug that is associated with NPs. It means that available methods are not able to differentiate whether the drug is adsorbed or retained in the polymeric matrix of the nanospheres, or if it is dissolved in the oil of the nanocapsules and/or adsorbed into the polymeric wall [97]. The mode of drug association mode with NPs is thus only estimated through comparative studies of zeta potential, release profiles, distribution of molar mass of the polymer, studies of adsorption and rate of association of the drug with nanostructures or even through the use of fluorescent probes [98]. Other techniques include differential scanning calorimetry, X-ray diffraction and infrared spectroscopy [67].

### 3.10. Pharmaceutical In Vitro Release Kinetics

According to Soppimath et al. [1], the release of drugs from polymeric NPs depends on the following factors [31,36], namely, the desorption of the drug from the surface of the particles and/or the polymeric matrix erosion, the diffusion of the drug through the nanosphere matrix or through the polymeric wall of the nanocapsules, or the combination of diffusion and erosion processes.

Methods, such as diffusion from dialysis bags and separation based on ultracentrifugation, low-pressure filtration, or ultrafiltration-centrifugation, have been used to describe the drug release from polymeric nanoparticles [99]. According to previous studies, the kinetics of drug release from nanospheres is generally in the form of an exponential (first order), possibly due to the drug diffusion from the polymeric matrix to the environment and/or erosion of the polymeric matrix, releasing the drug [31,100]. In the case of nanocapsules, the drug theoretically dissolved in the oily nucleus would be released from this vesicular structure upon its diffusion through the polymeric wall, presenting zero-order kinetics [101]. A work carried out by Calvo et al. [82], based on the similarity between the drug release profiles associated with nanocapsules and nanoemulsion, suggested that the nanocapsules’ polymeric walls do not influence the release process, as this is only affected by the partition of the drug between the oil droplets and the external aqueous medium.

## 4. From Ecotoxicology to Nanoecotoxicology

Ecotoxicology was defined by Truhaut in 1969 as “the branch of toxicology related to the study of the toxic effect, which causes natural or synthetic pollutants, for the constituents of ecosystems, animals (including humans), plants and microbials, in an integral context” [102]. Research in the field of ecotoxicology has developed rapidly due to environmental pollution caused by rapid industrial development [103,104]. Contrary to the approaches carried out by analytical chemistry, ecotoxicological tests integrate all toxic signs. Therefore it was proposed to add criteria based on toxicity to the current policies for the assessment of environmental risk [105]. Thus, policies have been developed concerning ecotoxicology assessment, which has become an important part of assessing environmental and ecological risks [103,105]. Ecotoxicology was developed mainly to assess aquatic toxicology (terrestrial ecotoxicological studies had less development than aquatic studies), in 1989 by van Straalen and Denneman [103]. Nanoecotoxicology stands for the toxicological effects that nanoparticles can induce on humans, animals, plants, fungi, and other microorganisms when released into the environment. Humans and animals are exposed to nanoparticles-based products through different ways, e.g., contaminated water, air, or even through the consumption of animals and/or plants that have been exposed to and accumulated nanomaterials. Despite the increase in knowledge about natural and synthetic nanoparticles, assessment of their potential environmental risk is essential before these particles are used in various products, which may later reach the environment. Currently, there is little data on the toxicity of nanomaterials for environmentally relevant species, limiting the quantitative risk assessment of nanomaterials [61,106].

### 4.1. Challenges in Nanoecotoxicology Research

In 2008, a publication by Behra and Krug in the section of “Nature Nanotechnology” indicated three main problems that must be resolved in the coming years [103]:

(i) The choice of nanoparticles for use in biological experiments and tests. It is necessary to determine the physicochemical properties, the capacity for aggregation and sedimentation, among other characteristics to identify the nanoparticles before, during and after the experiments;
(ii) The need to examine the pathway for the capture of synthetic nanoparticles by organisms in different environments (important for the behavior of synthetic nanoparticles in the food chain);
(iii) The set of organisms that can be used in experiments and measurement points that can be used.

### 4.2. Monitoring of Nanoparticles’ Toxicity

Analytical methods as described in Section 3, are instrumental to obtain information about the potential risks of polymeric NPs, so that an efficient action to ensure the safety of nanoparticles can be implemented [107]. Risks associated with the use of nanoparticles is based on their potential toxicity and interaction with living cells [59]. Attention should also be paid to the potential changes (chemical, physical) that the medium may induce to nanoparticles and on the degradation, which affects their bioavailability and in vivo behavior [107,108].

To ensure the quality of the results obtained from toxicity assessment, the methods used for monitoring the nanoparticles’ toxicity need to be able to detect very low concentrations of toxicological biomarkers and should avoid the potential interference of other compounds in the sample [67]. For the elaboration of an appropriate analytical procedure, several elements should be considered, namely:

- Sample treatment—A sampling of nanoparticle formulation and the laboratory procedures may change state of dispersion. Due to the unavailability of sufficiently sensitive portable equipment, it is not possible to identify fluctuations in situ [67].
- Separation of nanoparticles—It is often required to submit the samples to pre-fractionation by centrifugation or filtration in order to remove unwanted particles [109]. Centrifugation is a more efficient method for denser particles, while microfiltration is widely used due to its simplicity. Nanoparticles are deposited on a membrane by collision or by electrostatic attraction. Field flow fractionation may also be used to separate particles according to their size in relation to their diffusion coefficient. Size exclusion chromatography and capillary electrophoresis are other effective methods for separating and purifying nanoparticles according to their size [110].

## 5. In Vitro and Vivo Toxicological Studies

Nanoparticle drug delivery provides many advantages, because of their highly stable nature and ability to encapsulate different active ingredients. Thus, the issue of their nanotoxicity is highly significant [106]. Many properties of nanoparticles that may cause unexpected toxicities are equally interesting [111]. In order to prove a nanoparticle’s toxicity profile and response on the suitable animal model—a model that closely represents the pathophysiology of human disorder—preliminary effectiveness in vitro followed by in vivo tests should be analyzed [112].

Biocompatibility, biodegradability and non-toxicity are the main characteristics of polymeric NPs [111,113,114]. According to Maurya et al. (2019) [115], polymeric NPs are safe for human use and they can improve the bioavailability of the loaded drugs. The great importance that is given to these nanocarriers is due to their good stability and the ability to encapsulate a high amount of substances. A recent report has indicated that many biodegradable and non-biodegradable polymeric nanocarriers can be applied for oral drug delivery [115]. The successful study of curcumin-loaded polymeric NPs provided 5.6-fold higher oral bioavailability compared with pure curcumin. Similar to curcumin, in vivo study of silymarin from orally administered polymeric nanoemulsion has proved 4-fold higher efficiency than conventional silymarin suspension [115]. Natural polymers, such as chitosan, dextran, heparin, or hyaluronan have been widely used for drug delivery studies having their biodegradable, biocompatible and mucoadhesive properties [114]. Moreover, in order to synthesize more advanced and highly desired nanocarriers, the concept of biomimetic has been introduced in material design. In this case, suitable ligands are added to the carbon nanotubes surface or to fabricate chitosan nanoparticle [111].

Commonly used for drug delivery applications, biodegradable synthetic polymers, namely the saturated poly(α-hydroxy esters): poly(lactic acid) (PLA), poly(glycolic acid) (PGA), and poly(lactic-co-glycolide) (PLGA), have been approved by the US Food and Drug Administration and the European Medicine Agency [64,111] because of their safety profile, confirmed biocompatibility, low levels of immunogenicity and toxicity, as well as their biodegradation during in vivo studies [114]. It has been noticed that also polymeric nanogels show the minimum level of toxicity, stability in the presence of serum, and stimulus responsiveness, since they possess a high drug encapsulation capacity, tuneable size and are relatively easy to obtain. Therefore, they are widely used in biosensors, drug delivery, tissue engineering, and biomimetic materials design [113,114].

The special properties and features, such as size, surface charge, hydrophilicity and hydrophobicity, or even type of polymer, govern the potential application of polymeric NPs [112,114]. Importantly, the content of alcohols, amines and thiols provides a successful polymer functionalization. This is possible thanks to fast reaction kinetics, the stability of isocyanates toward radicals and the good yields, although their further use is limited by the toxicity of isocyanate or the instability of final mixtures containing isocyanate and polymer mixtures [113].

The cytotoxic profile of nanoparticles is commonly screened in vitro using colorimetric assays e.g., Alamar blue and 3-(4,5-dimethylthiazol-2-yl)-2,5-diphenyltetrazolium bromide (MTT), for the cell viability of selected cell lines. Cell viability over 70% is usually considered as a proof of low cytotoxicity of the tested nanoparticle formulation. The cytotoxicity of PLGA nanoparticles loading triterpenoids with potential anticancer activity has been tested against HepG2 (Human hepatoma cell line), Caco-2 (Human epithelial colorectal adenocarcinoma cell line) and Y-79 (Human retinoblastoma cell line) [59]. The natural and synthetic mixtures of oleanolic and ursolic acids were tested as free and loaded in PLGA nanoparticles in a concentration range from 2 to 32 micromol/L, showing that nanoparticles could significantly increase the cell viability when loading the triterpenoids into the particles.

PLGA nanoparticles loading dexibuprofen have been surface-modified with polyethylene glycol chains (PEGylated) in order to increase the retention time of particles in the ocular mucosa [52]. Dexibuprofen is the enantiomer of the non-steroidal anti-inflammatory ibuprofen, that has also been recommended for the treatment of inflammatory eye diseases. Cell viability studies in the human retinoblastoma cell line confirmed that PEGylated-PLGA nanospheres were less cytotoxic than free dexibuprofen, whereas ocular in vitro (chorioallantoic membrane test) and in vivo (Draize test) tolerance assays demonstrated the non-irritant character of the developed formulations. Pranoprofen is another non-steroidal anti-inflammatory drug (NSAID) considered safe for the anti-inflammatory treatment for strabismus and/or cataract surgery. It has been loaded into PLGA nanospheres with reportedly no cytotoxicity against Y-79 cell lines in therapeutic doses [51].

Based on the recent scientific reports, thanks to the ligand coupled onto the nanoparticle surface, the active targeting provides less toxicity to healthy tissues in the comparison with targeting ligands overexpressed on the tumor tissue [111]. For instance, ethoxy-(poly(ethylene glycol))-folic acid (FA-PEG) micelle consist of docetaxel (DTX) applied to cause a higher toxicity on FR-positive MCF-7 cells [116]. Likewise, the superiority of the polymer coating (e.g., PEG) is to control protein or peptide absorption via its hydrophilic chains that will also regulate cell behavior during contact [116]. On the other hand, the surface images of topical administration polystyrene NPs ex vivo and in vivo tests have shown NPs accumulation in the follicular openings. The first pass metabolism effect of drugs can be established by transdermal drug delivery system, hence, a lower amount of drug can be administered efficiently with reduced toxicity [116]. Fam et al. (2020) have described frequently used polymers, such as PEG, poly(2-oxazoline) (POx) and poly(zwitterions) in developing long-circulating NPs for drug delivery are also thoroughly discussed. The scientists have mentioned about the biomimetic approaches, including the cell-membrane camouflaging technique and functionalization for the design of stealth nano-delivery systems [117]. The use of polymeric NPs as chemotherapeutic drug delivery systems is often difficult because of a poor circulation stability and targeting inefficiency. To overcome these problems, Palanikumar et al. (2020) have obtained biocompatible and biodegradable pH responsive hybrid NPs. These nanosystems based on drug-loaded PLGA core were additionally coated by a crosslinked bovine serum albumin shell that was added to reduce interactions with serum proteins and macrophages. As a result, the drug-loaded NPs showed potent anticancer activity in vitro and in vivo while exhibiting no toxicity to healthy tissue [118].

Currently, the influence of polymer NPs is significant in medicine, however, their clinical use needs to be critically controlled, due to the potential toxicity of their components, although polymers are mostly biodegradable and ensure the easy excretion of their oligomers through common metabolic pathways [119]. The inherent toxicity of all components of the formulation (i.e., drugs, polymers and other excipients) have to be screened for toxicity [120].

## 6. Conclusions

Understanding the physicochemical behavior of polymeric NPs is the subject of numerous researches, however one of the main difficulties encountered in their characterization is their nano size. The physicochemical characterization of these drug carriers is only feasible through the combination of several analytical techniques. One of the biggest challenges, which still persists, is the elucidation of drug association mechanisms to polymeric NPs. Several advances have been already achieved, both in the accumulation of information related to the physicochemical phenomena involved and in relation to the development of more stable polymeric NP formulations, which can broaden the prospects for clinical use of these systems. The study of nanoparticles and nanoecotoxicology is necessary to continue the development of efficient nanocarriers, showing no risk for the environment or human health in their potential applications. Particularly with chemical metrology in mind, it will be possible to obtain quantitative and qualitative measurements that help to establish methods, principles, and procedures that guarantee results with analytical quality.

## Figures and Tables

**Table 1:** Examples of drugs/bioactive ingredients loaded in polymeric nanoparticles.

Type of Polymers	Formulated Drug/Bioactive	Type of Polymeric Nanoparticles/Method	Applications Purpose	Ref.
PCL, PLA, PLGA	Coumarin-6 (C-6)	nanospheres; C-6-loaded polymeric core-shell NPs (polymeric core-multilayer polyelectrolyte shell NPs), obtained by prepared by the spontaneous emulsification solvent evaporation method	drug delivery, theranostics, or bioimaging	[9]
PLGA	Rapamycin	nanospheres; Rapamycin-loaded polysorbate 80-coated PLGA NPs	anti-glioma activity	[10]
AcDex	Hyperforin	nanospheres; Hyperforin-loaded AcDex-based NPs formulated via single emulsion/solvent evaporationusing ethyl acetate and water	anti-inflammatory activity	[11]
PLGA	Fenofibrate (Feno)	nanospheres; PLGA-Feno NPs	diabetic retinopathy, neovascular age-related macular degeneration (ocular neovascularization)	[12]
biopolymer of PCL	Amphotericin B (Amp B)	nanocapsules; PCL-NCs loaded with Amp B, obtained by nanoprecipitation method	anti-leishmanial (*Leishmania* infections), anti-fungal	[13]
anionic copolymers based on methacrylic acic and methyl methacrylate (Eudragit L 100)	Fenofibrate (FF)	nanocapsules; FF-loaded-Eudragit L 100 NCs, obtained by nanoprecipitation method	undefined oral delivery	[14]
PLGA, PCL	Ciprofloxacin	nanocapsules; ciprofloxacin-loaded PLGA NCs, obtained by nanoprecipitation method	in situ tissue regeneration and accelerated healing, anti-inflammatory activity	[15]
PLGA	Curcumin (Cur)	nanocapsules; Cur-loaded PLGA NCs	antibacterial activity, pancreatic cancer	[16,17]
F108: PEG-PPG-PEG	Curcumin (Cur)	colloidal nanocapsules; Cur-loaded PEG-PPG-PEG NCs	anticancer	[18]
PEG	Pegademase bovine	colloidal nanocapsules; Pegademase bovine-loaded PEG NCs	severe combined immunodeficiency disease	[17,19]
PCL-PEG-PCL	Paclitaxel (PTX)	nanocapsules; PTX-loaded PCL-PEG-PCL NCs	lung cancers in combinationwith chrono-modulatedchemotherapy	[20]
PLGA-PEG	Paclitaxel (PTX)	nanocapsules; PTX-loaded PLGA-PEG NCs	breast, pancreatic andovarian and brain cancers	[20]
**Eudragit^®^ RS100, Eudragit^®^ L100-55, Eudragit^®^ EPO, PCL, polylactide, PLGA**	**Essential Oils**	**EO based-nanoparticles by nanoprecipitation method**	**antioxidant/antimicrobial**	[21]
PCL	*Cymbopogon martini Roxb.* (Palmarosa oil)	nanocapsules; Palmarosa oil-loaded PCL NCs	antioxidant, antimicrobial	[22]
Eudragit^®^ L100-55	*Thymus vulgaris L.* (Thyme oil)	nanocapsules; Thyme oil-loaded Eudragit^®^ L100-55 NCs	antioxidant	[23]
Eudragit^®^ RS100	*Citrus bergamia Risso.* (Bergamot oil)	nanocapsules; Bergamot oi-loaded Eudragit^®^ RS100 NCs	antimicrobial	[24]
Eudragit^®^ RS100	*Citrus sinensisL.* (Orange oil)	nanocapsules; Orange oil-loaded Eudragit^®^ RS100	antimicrobial	[24]
Eudragit^®^ EPO	*Rosmarinus officinalis L.* (Rosemary oil)	nanocapsules; Rosemary oil-loaded Eudragit^®^ EPO NCs	antioxidant	[25]
Eudragit^®^ EPO	*Lavandula dentata L.* (Lavender oil)	nanocapsules; Lavender oil-loaded Eudragit^®^ EPO NCs	antioxidant	[25]
PCL	Geraniol	nanocapsules; Geraniol-loaded PCL NCs	antioxidant, antimicrobial	[22]

**Abbreviations:** AcDex—acetalated dextran; F108—poly(ethylene oxide)-*block*-poly(propylene oxide)-*block*-poly(ethylene oxide); NCs—nanocapsules; NPs—nanoparticles; PCL—poly(ε-caprolactone); PEG—poly(ethylene glycol); PLA—poly(lactic acid); PLGA—poly(lactide-co-glycolide).

**Table 2:** Different methods for the production of polymeric nanoparticles.

Polymeric Nanoparticles	Production Methods
Nanospheres	Solvent evaporation Emulsification/solvent diffusion Nanoprecipitation Emulsification/reverse salting-out
Nanocapsules	Nanoprecipitation
